# Incompatibility of chemical protein synthesis inhibitors with accurate measurement of extended protein degradation rates

**DOI:** 10.1002/prp2.359

**Published:** 2017-09-29

**Authors:** Christina Chan, Philip Martin, Neill J. Liptrott, Marco Siccardi, Lisa Almond, Andrew Owen

**Affiliations:** ^1^ Department of Molecular and Clinical Pharmacology The University of Liverpool 70 Pembroke Place, Block H (first floor) Liverpool L69 3GF United Kingdom; ^2^ Simcyp (a Certara company) Blades Enterprise Centre John Street Sheffield S2 4SU United Kingdom

**Keywords:** cytotoxicity, protein degradation rate, protein synthesis inhibitor

## Abstract

Protein synthesis inhibitors are commonly used for measuring protein degradation rates, but may cause cytotoxicity via direct or indirect mechanisms. This study aimed to identify concentrations providing optimal inhibition in the absence of overt cytotoxicity. Actinomycin D, cycloheximide, emetine, and puromycin were assessed individually, and in two‐, three‐, and four‐drug combinations for protein synthesis inhibition (IC_50_) and cytotoxicity (CC_50_) over 72 h. Experiments were conducted in HepG2 cells and primary rat hepatocytes (PRH). IC_50_ for actinomycin D, cycloheximide, emetine, and puromycin were 39 ± 7.4, 6600 ± 2500, 2200 ± 1400, and 1600 ± 1200 nmol/L; with corresponding CC_50_ values of 6.2 ± 7.3, 570 ± 510, 81 ± 9, and 1300 ± 64 nmol/L, respectively, in HepG2 cells. The IC_50_ were 1.7 ± 1.8, 290 ± 90, 620 ± 920, and 2000 ± 2000 nmol/L, with corresponding CC_50_ values of 0.98 ± 1.8, 680 ± 1300, 180 ± 700, and 1600 ± 1000 (SD) nmol/L, respectively, in PRH. CC_50_ were also lower than the IC_50_ for all drug combinations in HepG2 cells. These data indicate that using pharmacological interference is inappropriate for measuring protein degradation over a protracted period, because inhibitory effects cannot be extricated from cytotoxicity.

**Abbreviations**
CC_10_: cytoxicity concentration at 10% of maximum (90% cell viability)
CC_50_: cytoxicity concentration at 50% of maximum (50% cell viability)
DDIs: drug–drug interactions
FICs: fractional inhibitory concentrations
GST: glutathione *S*‐transferase
HBSS: Hank's balanced salt solution
HepG2: hepatocellular carcinoma cell line
*k*_deg_: degradation rate constant
MTT: 3‐(4,5‐dimethylthiazol‐2‐yl)‐2,5‐diphenyltetrazolium bromide
PBPK: physiologically based pharmacokinetic
PRH: primary rat hepatocyte

## Introduction

Protein abundance in a cellular system is a balance between the rate of synthesis and degradation. The ability of the cell to remove and replenish proteins in a dynamic state of constant turnover is paramount to maintaining essential cellular functions. While rates of protein synthesis are readily measurable by time‐course experiments utilising radioisotopes and protein quantification, the rate of degradation (*k*_deg_) is often more difficult to determine especially in vivo (Millward et al. 1981; Pratt et al. 2002). This is due to the complex interplay between different protein degradation mechanisms and paucity in understanding the causal signalling mechanisms initiating specific protein degradation. Protein degradation is commonly quantified as half‐life, the time taken for protein to decrease by half (Zhou 2004; Belle et al. 2006; Zhang et al. 2007). This variable is interchangeable with *k*_deg_ by the following equations assuming first‐order decay kinetics (Belle et al. 2006), where *N* is the protein intensity, *k* is the decay rate constant (and *–k* represents *k*_deg_), and *t*_1/2_ is the half‐life:

(1) $$N=N_{0}e^{-kt}$$

(2) $$\text{ln}\left(N\right)-\;\text{ln}\left(N_{0}\right)=-kt\;\Rightarrow \;t_{\frac{1}{2}}=\;\frac{\ln\left(2\right)}{k}$$

(3) $$-k=\left(\ln\left[N\right]-\ln\left[N_{0}\right]\right)÷t$$

Physiologically based pharmacokinetic (PBPK) modelling can be used to predict the magnitude and dynamics of drug–drug interactions (DDIs), allowing the investigation of optimal timings for washout periods or switching of drug regimens in clinical practice. Such approaches require robust drug and system parameters (Jamei et al. 2009; Rostami‐Hodjegan 2012). Clearly, *k*_deg_ is a critical system parameter for the simulation of time‐dependent DDIs, such as those mediated by mechanism‐based inhibition or induction (Venkatakrishnan and Obach 2007; Almond et al. 2009).

Several sources have highlighted the lack of accurate *k*_deg_ data for metabolising enzymes and transporter proteins as important sources of error in DDI prediction (Obach et al. 2007; Wang 2010). Despite its well‐established impact, there is large disparity in the literature for the *k*_deg_ of specific proteins and different values are used for the same enzyme across different studies, resulting in inconsistent predictions (Ghanbari et al. 2006; Yang et al. 2008; Wang 2010; Yeo et al. 2011). Proteins have widely varied half‐lives, ranging from minutes to several days, and protein turnover is tightly regulated through multiple molecular mechanisms. Apart from the importance in PBPK, further characterisation of *k*_deg_ for specific proteins is required for better understanding of cell signalling processes involved in both normal and dysfunctional diseased cell states, thus studies of protein turnover are used in many different areas of cellular and molecular biology.

Traditional methods of protein degradation measurement and derivation of *k*_deg_, fall into two experimental designs: (1) quantifying the amount of a specific protein before and after a cell perturbation then measuring the difference in protein abundance and time between the initial and new steady‐state; or (2) quantifying changes in protein abundance by kinetic, time‐course experiments (Alvarez‐Castelao et al. 2012). The kinetic approach is based on an initial cell treatment with protein synthesis inhibitors followed by the quantification of changes in protein content over time by immunoblotting (Dai et al. 2013). Traditional methods of measuring protein degradation generally utilise low level incorporation of radiolabelled amino acids in the form of pulse‐chase analysis, often involving the use of protein synthesis inhibitors to eliminate reincorporation (Zhou 2004; Doherty et al. 2009). The more recent approaches focus on simultaneously measuring the rates of a large number of proteins. For example, stable isotope labelling by amino acids (SILAC) in cell culture followed by mass‐spectrometry (MS) as a common proteomics‐based method for measuring protein turnover rates (Mann 2006; Doherty et al. 2009; Fierro‐Monti et al. 2013; Takahashi et al. 2017) and isobaric tag for relative and absolute quantification (iTRAQ) are also used (Jayapal et al. 2010). The focus of this study was on the more traditional methods of measuring protein degradation utilising protein synthesis inhibitors for pharmacological interference.

The aim of this study was to find a suitable protein synthesis inhibitor or drug combination that provided maximum protein synthesis inhibition with minimum cytotoxicity for subsequent use in measuring protein degradation rates. The four selected inhibitors actinomycin D, cycloheximide, emetine, and puromycin were assessed alone and in combination to determine their suitability for protein degradation studies. Leucine incorporation assays and standard 3*‐*(4,5‐dimethylthiazol‐2‐yl)*‐*2,5‐diphenyltetrazolium bromide (MTT) assays were employed to determine the level of protein synthesis inhibition and cytotoxicity, respectively, across a range of drug concentrations in immortalised hepatic cell line and primary hepatocytes. Two‐drug combinations were tested for synergy by the modified fixed‐ratio isobologram method. Combinations of three and four inhibitors were assessed at subcytotoxic concentrations of each inhibitor.

## Materials and Methods

### Materials

Dulbecco's modified eagle medium (DMEM), fetal bovine serum (FBS), trypsin‐EDTA solution, Hank's balanced salt solution (HBSS), thiazolyl blue tetrazolium (TBT), and protein synthesis inhibitors (actinomycin D (A4262), emetine dihydrochloride hydrate (E2375), and puromycin dihydrochloride (P7255)) were purchased from Sigma‐Aldrich (Dorset, UK). HepG2 cells were purchased from American Tissue Culture Collections (ATCC, Virginia). Cryopreserved primary rat hepatocytes, William's E media, plating cocktail, maintenance cocktail, Geltrex^®^ matrix, and collagen I coated plates were purchased from Invitrogen Ltd (Paisley, UK). Cycloheximide (ab120093) was purchased from Abcam (Cambridge, UK). l‐Leucine [4,5‐^3^H] (MT‐672E) was obtained from Moravek (California). The CellTiter‐Glo cell viability assay and the GSH‐Glo glutathione assay were purchased from Promega (Southampton, UK).

### Cell line culture

HepG2 cells were grown in DMEM medium supplemented with 10% FBS solution and were discarded beyond passage 20. The media was changed every 48 h and cells were cultured until 80–90% confluence in a 37°C 5% CO_2_ humidified incubator. Cell counts were carried out by a Nucleocounter (Chemometec, Denmark).

### Primary rat hepatocyte culture

Primary rat hepatocytes (PRH) were purchased from Invitrogen (Paisley, UK), isolated from male Sprague–Dawley rats at 9 weeks old (Lot. RS745). Cryopreserved PRH were thawed in a 37°C water bath for approximately 2 min until contents were around 90% thawed. Once thawed, the hepatocytes were added to 50 mL of prewarmed plating media (William's E media without phenol red supplemented with 5% FBS, 1 *μ*mol/L dexamethasone, 1% solution of penicillin/streptomycin, 4 *μ*g/mL bovine insulin, 2 mmol/L GlutaMAX™, and 15 mmol/L HEPES (CHRM^®^ supplement A), and centrifuged for 3 min at 55*g* at 18°C and the supernatant fraction discarded. The hepatocytes were then resuspended in plating media at 1 × 10^6^ cells per ml density.

The cell viability of primary human hepatocytes was calculated using the Chemometec NucleoCounter^®^ NC‐100™ according to the manufacturer's protocol. Cells were seeded in collagen coated plates and were incubated for 5 h at 37°C with 5% CO_2_ and 95% humidity. After 5 h incubation, plating media was discarded and replaced with 0.022 mg/mL of Geltrex^®^ Matrix in maintenance media (William's E media supplemented with 0.1 *μ*mol/Ldexamethasone, 0.5% penicillin/streptomycin 6.25 *μ*g/mL human recombinant insulin, 6.25 *μ*g/mL human transferrin, 6.25 ng/mL selenous acid, 1.25 mg/mL BSA, 5.35 *μ*g/mL linoleic acid, 2 nmol/L GlutaMAX™, and 15 mM HEPES). After incubation overnight, media containing Geltrex^®^ was removed and replaced with varying drug concentrations and controls in maintenance media.

### Measuring protein synthesis inhibition by [^3^H]‐leucine incorporation

HepG2 cells were seeded at 2 × 10^5^ cells per well in DMEM supplemented with 10% FBS and the plates were incubated overnight at 37°C to allow cells to adhere. PRH cells were seeded in collagen coated 24‐well plates at a density of 2 × 10^5^ cells per well. Old media was removed and replaced with 0–100 *μ*mol/L of protein synthesis inhibitors dissolved in DMEM with 10% FBS for HepG2 cells or maintenance media for PRH and incubated for 72 h in a 37°C humidified incubator. In the last 2 h of incubation, cells were pulsed with 2 *μ*Ci of [^3^H]‐leucine without removing the inhibitor. After 2 h, the media containing [^3^H]‐leucine was removed by aspiration and the cells were washed with HBSS before removal from well by trypsinisation. HepG2 cells were then harvested onto a filtermat using a TomTec cell harvester. The filtermat was sealed in a sample bag with melt‐on scint and the level of protein synthesis was determined by the level of [^3^H]‐leucine incorporation measured using a MicroBeta detector (Perkin‐Elmer, Cambridge, UK). PRH cells were transferred to scintillation vials and radioactivity was determined using QuantaSmart™ software on a Tri‐Carb scintillation counter (Perkin‐Elmer).

### Measuring cell viability by standard MTT Assays

Standard MTT assays were performed on HepG2 and PRH cells to measure cell viability. 2 × 10^4^ cells per well of HepG2 were seeded into 96‐well plates in DMEM with 10% FBS and left overnight in a 37°C humidified incubator to allow cells to adhere to the plate. PRH were seeded in collagen coated 96‐well plates at a density of 2 × 10^4^ cells per well. Old media was removed and replaced with 0–300 *μ*mol/L of protein synthesis inhibitors and incubated for 72 h. A vehicle control and control with no drug was included. A quantity of 20 *μ*L of 5 mg/mL TBT in HBSS was added to each well and incubated for 2 h. A quantity of 100 *μ*L lysis buffer (50% v/v dimethylformahyde and 20% v/v sodium dodecyl sulphate) was added to each well and the plate was incubated overnight at 37°C. The absorbance was quantified at 570 nm by a Tecan GENios micoplate reader (Germany).

### Single protein synthesis inhibitor analysis

The protein synthesis inhibitors actinomycin D, cycloheximide, emetine, and puromycin were analysed individually in HepG2 and PRH cells. Actinomycin D was incubated 0–10 *μ*mol/L and 0–0.039 *μ*mol/L and puromycin at 0–20 *μ*mol/L and 0–5 *μ*mol/L for leucine incorporation assays and MTT cytotoxicity assays, respectively. Cycloheximide was incubated at 0–300 *μ*mol/L and emetine at 0–30 *μ*mol/L for both leucine incorporation and MTT assays.

### Two‐drug combination fixed‐ratio isobologram analysis

The effects of two‐drug combinations on HepG2 cells were assessed by the modified fixed‐ratio isobologram protocol, which detects synergy, additivity, or antagonism between a pair of drugs (Fivelman et al. 2004). Stock solutions of the drugs were prepared at 10 mmol/L in sterile distilled water. Concentration–response assays were carried out to obtain the IC_50_ and CC_50_ of the individual drugs by leucine incorporation and standard MTT assays, respectively. For the six two‐drug combinations, the drug dilutions were made to allow the IC_50_ or CC_50_ to fall at about the fourth threefold serial dilution. The dilutions of each of the two drugs in each combination were prepared in seven fixed ratios 6:0, 5:1, 4:2, 3:3, 2:4, 1:5, and 0:6. These mixtures were then serially diluted threefold in quadruplicates to generate a range of eight concentrations for each condition. Protein synthesis inhibition and cell viability assays were conducted as described above to generate a concentration–response curve to calculate the IC_50_ and CC_50_ for drugs A and B in each mixture. The fractional inhibitory concentrations (FICs) were calculated using Equation (4), (5), and 6 (Gorka et al. 2013):

(4) $${\text{FIC}}_{A}=\frac{{\text{IC}}_{50}\;\text{or}\;{\text{CC}}_{50}\;\text{of drug A in combination}}{{\text{IC}}_{50}\;\text{or}\;{\text{CC}}_{50}\;\text{of drug A alone}}$$

(5) $$\text{FI}\;C_{B}=\frac{I\;C_{50}\text{or}\;{\text{CC}}_{50}\text{of drug B in combination}}{I\;C_{50}\text{or}\;{\text{CC}}_{50}\text{of drug B alone}}$$

(6) $${\text{FIC}}_{\mathrm{index}}={\text{FIC}}_{A}+{\text{FIC}}_{B}$$

Isobologram curves were generated by plotting FIC_A_ versus FIC_B._ FIC_index _= 1 was taken as indicative of an additive effect between drugs A and B, FIC_index_<1 indicative of synergy and FIC_index_>1 indicative of antagonism.

### Three‐ and four‐drug combination analysis

Three‐drug combinations: actinomycin D, cycloheximide, and emetine; actinomycin D, puromycin, and emetine; actinomycin D, puromycin, and cycloheximide; and puromycin, cycloheximide and emetine, and four‐drug combination: actinomycin D, puromycin, cycloheximide, and emetine were assessed at subcytotoxic concentrations of each drug (determined from the single drug incubation experiments) in HepG2 cells. The three‐ and four‐drug combinations were made up at the CC_10_ concentrations and measured for level of protein synthesis inhibition by [^3^H]‐leucine incorporation and assessed for cytotoxicity by several different toxicity assays.

#### Standard MTT assay

Standard MTT assays were performed on the three‐ and four‐drug combinations using methods described above in HepG2 cells. Further toxicity assays (CellTiter‐Glo^®^, GSH‐Glo™ glutathione, and trypan blue exclusion) were performed on these combinations to confirm the robustness of MTT assays as a measure of cell viability.

#### CellTiter‐Glo^®^ luminescent cell viability assay

A CellTiter‐Glo^®^ luminescent cell viability assay was performed on the above drug combinations following 72 h incubation in HepG2 as described in the manufacturer's protocol. Cells were seeded at 2 × 10^4^ cells per well in DMEM with 10% FBS. The assay measures the amount of ATP present that indicates the presence of metabolically active viable cells.

#### GSH‐Glo™ glutathione assay

GSH‐Glo™ glutathione assays were performed on the above drug combinations following 72 h incubation in HepG2 cells according to the manufacturer's protocol. Cells were seeded at 1 × 10^4^ cells per well in DMEM with 10% FBS. The assay measures the conversion of a luciferin derivative into luciferin in the presence of glutathione and glutathione *S*‐transferase (GST) as an indication of oxidative stress.

#### Trypan blue exclusion

HepG2 cells were seeded at 5 × 10^4^ cells per well in DMEM +10% FBS and incubated with the three‐ and four‐drug combinations for 72 h. Following incubation, the cells were washed with HBSS solution and trypsinised for 5 min before being transferred in suspension to Eppendorf tubes. A quantity of 10 *μ*L of cell suspension was added to 10 *μ*L of trypan blue solution and placed on a Countess™ slide. Cell viability was calculated using a Countess™ automated cell counter (LifeTechnologies, UK).

#### Data analysis

The IC_50_ (concentration causing 50% protein synthesis inhibition), CC_50_ (concentration causing 50% cell viability), and CC_10_ (concentration causing 90% cell viability) were calculated by nonlinear regression of drug concentration versus leucine incorporation and MTT concentration–response graphs, respectively, using Graphpad Prism 3 software. The IC_50_ and CC_50_ values derived from the single inhibitor analyses were used for subsequent fixed‐ratio isobologram two‐drug combination analyses.

## Results

### Single protein synthesis inhibitor

**Figure 1:**
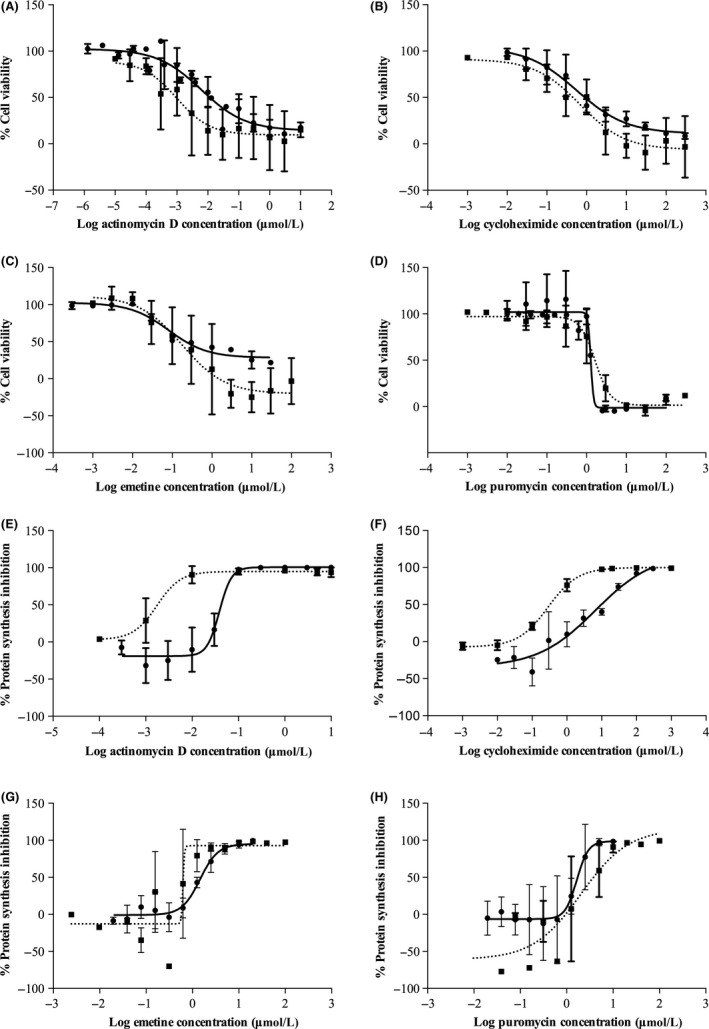
IC_50_ and CC_50_ of the four individual protein synthesis inhibitors in HepG2 and primary rat hepatocytes (PRH). (A–D) Cell viability was measured by standard MTT assay and expressed as viability as a percentage of untreated control. (E–H) Protein synthesis inhibition across different concentrations of inhibitors was measured by [^3^H]‐Leucine incorporation assay and shown as percentage of inhibition of control. Dotted line shows PRH and solid line for HepG2 cells. Dose–response curves were produced by Prism software and IC_50_ and CC_50_ values were calculated from linear regression models. Data are shown as mean ± SD from *n* = 3 independent experiments.

The mean ± SD CC_50_ for the four protein synthesis inhibitors actinomycin D, cycloheximide, emetine, and puromycin were found at 6.2 ± 7.3, 570 ± 510, 81 ± 9, and 1300 ± 64 nmol/L, respectively, in HepG2 cells and 0.98 ± 1.8, 680 ± 1300, 180 ± 700, and 1600 ± 1000 nmol/L, respectively, in PRH. The IC_50_ were 39 ± 7.4, 6600 ± 2500, 2200 ± 1400, and 1600 ± 1200 *μ*mol/L, respectively, in HepG2 and 1.7 ± 1.8, 290 ± 90, 620 ± 920, and 2000 ± 2000 nmol/L, respectively, in primary rat hepatocytes. The IC_50_ and CC_50_ concentrations were calculated from concentration–response graphs as shown in Figure 1. The CC_50_ concentrations were lower compared to corresponding IC_50_ values for all four inhibitor drugs except cycloheximide in PRH; this indicates that the inhibitors were more effective in generating cell death than protein synthesis inhibition and thus unsuitable for further protein degradation studies.

**Figure 2:**
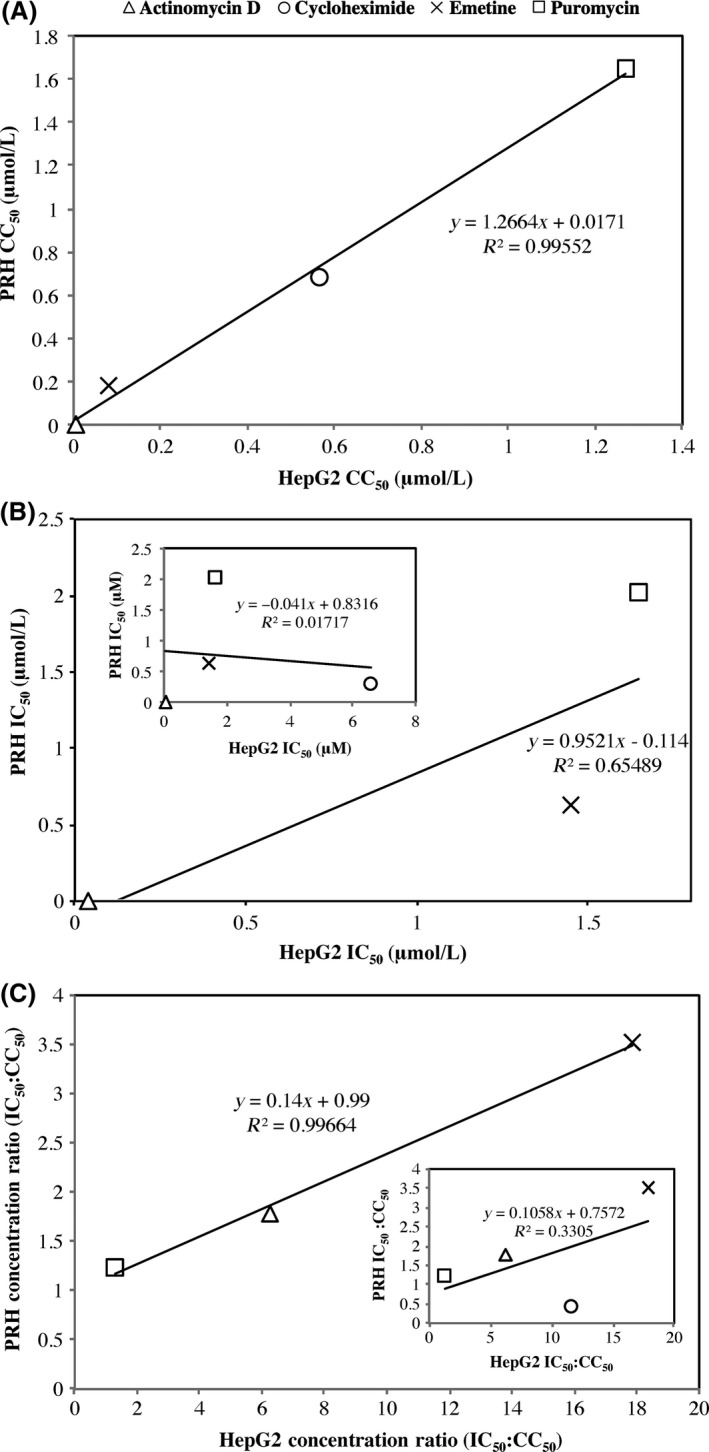
Linear regression analysis of IC_50_ and CC_50_ between HepG2 and PRH cell types. (A) shows linear regression between CC_50_ values of the four protein synthesis inhibitor drugs for the different cell types. (B) shows linear regression between IC_50_ values with cycloheximide omitted but shown in inset graph, of the two cell types. (C) shows IC_50_:CC_50_ ratio of HepG2 and PRH cell types omitting cycloheximide. Cycloheximide is included in the inset graph.

Figure 2 shows linear regression between the IC_50_ and CC_50_ values derived from HepG2 and cryopreserved PRH cells. Figures 2A–C show linear relationships between the IC_50_ and CC_50_ between HepG2 and PRH cells for actinomycin D, emetine, and puromycin. Cycloheximide fit in the linear relationship for cytotoxicity but not for protein synthesis inhibition.

### Two‐drug protein synthesis inhibitor combinations

**Figure 3:**
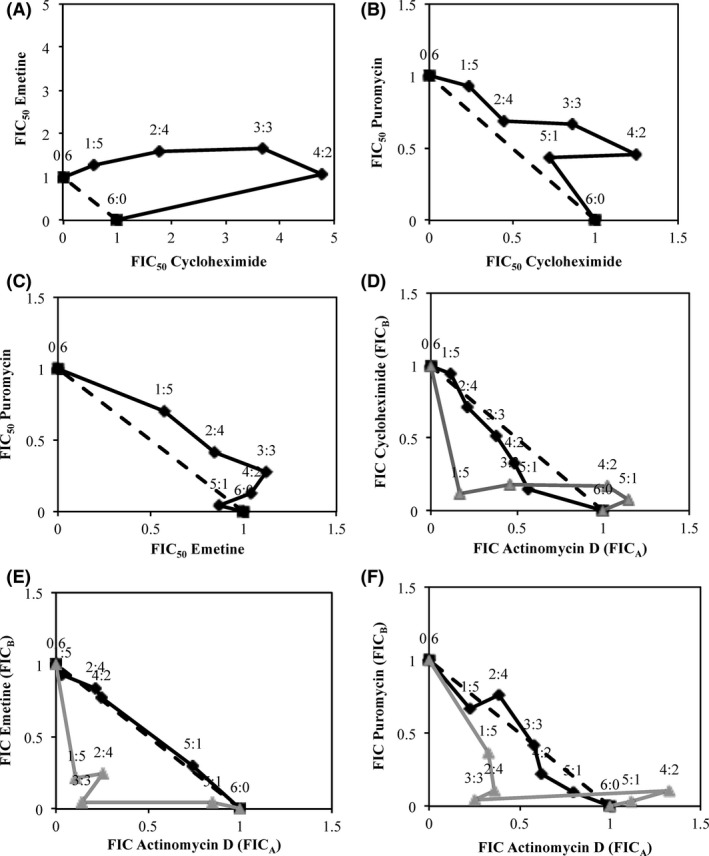
Isobolograms generated based on CC_50_ and IC_50_ values showing the interaction between protein synthesis inhibitor pairs. Six combinations of inhibitor pairs are shown in (A–F) The dotted line corresponds to the predicted curve if drug pairs showed an additive effect. The black line corresponds to drug pair interactions for protein synthesis inhibition. The grey line shows drug pair interactions for cytotoxicity. FIC_A_ and FIC_B_ correspond to the fractional inhibitory concentrations of the first and second drugs in each drug pair listed. Cytotoxicity analysis was not performed for cycloheximide–emetine, cycloheximide–puromycin, and emetine–puromycin drug pairs (A–C) as these showed strong antagonism for protein synthesis inhibition. *N* = 4 independent experiments were carried out in HepG2 cells.

The fixed‐ratio isobologram method was employed to assess additivity, synergy, or antagonism in both protein synthesis inhibition and cytotoxicity between drug pairs. Six combinations of drug pairs for the four inhibitors were analysed. The combinations cycloheximide and emetine, cycloheximide and puromycin, and emetine and puromycin showed antagonism for protein synthesis inhibition at all ratios (as shown in Fig. 3A–C) and were therefore deemed to be unsuitable for protein degradation studies. As such, isobolograms to assess cytotoxicity were not carried out for these combinations. Actinomycin D and emetine showed additivity (no interaction) between the drugs for protein synthesis inhibition and synergy for cytotoxicity, indicating that actinomycin D and emetine did not increase protein synthesis inhibition in combination but did display higher cytoxicity. As such, this combination was also deemed unsuitable for measuring protein degradation rates. Actinomycin D and cycloheximide, and actinomycin D and puromycin did show synergy for protein synthesis inhibition at some ratios. This combination also displayed strong synergy for cytotoxicity at most ratios. Interestingly, at ratios of 5:1 and 4:2 for actinomycin D: cycloheximide and actinomycin D: puromycin, these combinations were synergistic for protein synthesis inhibition and antagonistic for cytotoxicity as seen in Figure 3D and F, respectively. However, despite the synergy for protein synthesis inhibition and antagonism for cytotoxicity at these ratios, the CC_50_ values for these drug pairs alone and in combination were still lower than the IC_50_ values and thus cytotoxicity was observed at lower concentrations than those required to inhibit protein synthesis. The CC_50_ concentrations for actinomycin D in combination with cycloheximide at 5:1 and 4:2 ratios were 12 and 14 nmol/L and the corresponding IC50 concentrations were 28 and 35 nmol/L, respectively. The CC_50_ values for cycloheximide in combination with actinomycin D at 5:1 and 4:2 ratios were 26 and 12 nmol/L and the corresponding IC_50_ concentrations were 2500 and 1300 nmol/L respectively. For the combination actinomycin D and puromycin, the CC_50_ concentrations for actinomycin D at 5:1 and 4:2 ratios were 9.8 and 8.1 nmol/L and the corresponding IC_50_ concentrations were 16 and 21 nmol/L respectively. As for puromycin, the CC_50_ values at 5:1 and 4:2 ratios were 60 and 20 nmol/L and the corresponding IC_50_ concentrations were 690 and 360 nmol/L respectively.

### Three‐ and four‐drug combination analysis

**Figure 4:**
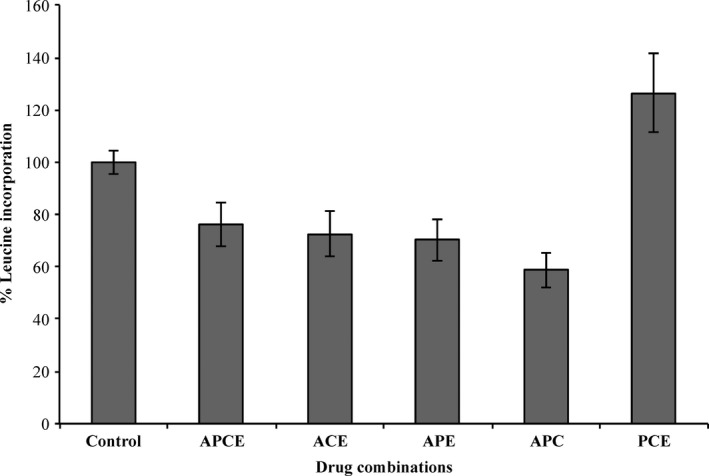
The level of [^3^H]Leucine incorporation for three‐ and four‐inhibitor combinations at subcytotoxic concentrations (CC_10_). Leucine incorporation assays were carried out in HepG2 cells and the percentage of incorporation compared to control was calculated. Combination APCE corresponds to actinomycin D, puromycin, cycloheximide, and emetine; ACE to actinomycin D, cycloheximide, and emetine; APE to actinomycin D, puromycin, and emetine; APC to actinomycin D, puromycin, and cycloheximide; and PCE to puromycin, cycloheximide, and emetine. Data are shown as mean ± S.D from *n* = 3 independent experiments.

**Figure 5:**
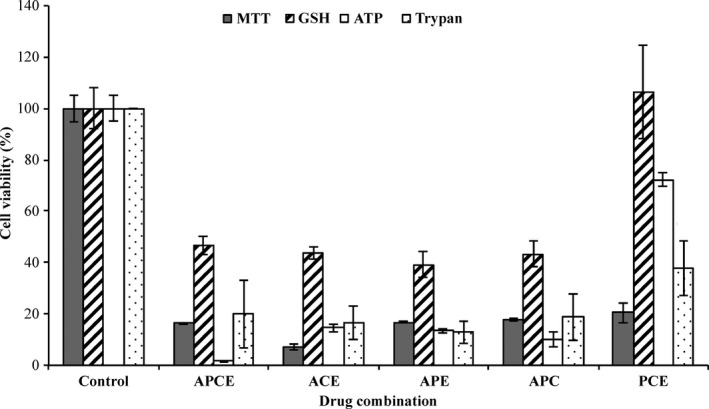
Measuring cytotoxicity for the three‐ and four‐inhibitor combinations at subcytotoxic concentrations. Three‐ and four‐inhibitor combinations were prepared at CC_10_ concentrations. A range of cytotoxicity assays including standard MTT, GSH, ATP, and trypan blue exclusion assays were conducted on HepG2 cells. APCE corresponds to actinomycin D, puromycin, cycloheximide, and emetine; ACE to actinomycin D, cycloheximide, and emetine; APE to actinomycin D, puromycin, and emetine; APC to actinomycin D, puromycin, and cycloheximide; and PCE to puromycin, cycloheximide; and emetine. Data are shown as mean ± SD from *n* = 3 independent experiments.

The four inhibitors individually and the two‐drug combinations displayed high cell death. Three‐ and four‐drug combinations at subtoxic concentrations (CC_10_ of each when incubated alone) were, therefore, assessed to investigate whether protein synthesis inhibition could be achieved at concentrations lower or equal to those causing cytotoxicity. The CC_10_ (90% cell viability concentration) were calculated for each drug to be 0.17, 24, 7.0, and 110 nmol/L for actinomycin D, cycloheximide, emetine, and puromycin, respectively, in HepG2 cells. As mentioned previously, the inhibitors alone displayed a lower concentration for CC_50_ than IC_50_ indicating that they were more effective in generating cell death than inhibiting protein synthesis. The four‐drug combination showed a high 76% protein synthesis incorporation (thus low inhibition) and high cytotoxicity across all cytotoxicity assays as seen in Figures 4 and 5. Three‐drug combinations: actinomycin D, cycloheximide, and emetine; actinomycin D, cycloheximide, and puromycin; and actinomycin D, puromycin and emetine also demonstrated low protein synthesis inhibition with high cytotoxicity, also seen in Figures 4 and 5. Although puromycin, cycloheximide, and emetine gave low cell death across the assays, it was also ineffective at inhibiting protein synthesis inhibition, as shown in Figure 4, where level of leucine incorporation is higher than control. Overall, three‐ and four‐drug combinations of these protein synthesis inhibitors were deemed to be unsuitable for further protein degradation studies even at low concentrations.

## Discussion

The use of protein synthesis inhibitors is the most common method for measuring protein degradation rates and has been documented by many sources over four decades (Goldberg and Dice 1974; Curfman et al. 1980; Princiotta et al. 2003; Zhou 2004; Belle et al. 2006; Delgado‐Vega et al. 2012; Chistyakov et al. 2014). The more recent approaches focus on simultaneously measuring the rates of a large number of proteins. For example, (SILAC) in cell culture followed by (MS) as a common proteomics‐based method for measuring protein turnover rates (Mann 2006; Doherty et al. 2009; Fierro‐Monti et al. 2013; Takahashi et al. 2017) and (iTRAQ) are also used (Jayapal et al. 2010). However, the wide application of these proteonomic approaches are limited by cost and complexity. The focus of this study was on the more simple traditional methods of measuring protein degradation utilising protein synthesis inhibitors for pharmacological interference. The aim of this study was to define inhibitor concentrations (single or combinations) that provide maximum protein synthesis inhibition with minimum cytotoxicity that could then be used in subsequent experiments to accurately estimate endogenous degradation rates.

For this study, four protein synthesis inhibitors actinomycin D, cycloheximide, emetine, and puromycin were selected based on their different mechanisms of action and previous use in biomedical research. Actinomycin D (Sobell 1985) intercalates DNA forming a stable complex with deoxyguanosine residues, thus blocking movement of RNA polymerase and subsequently transcription. Cycloheximide binds the 60S ribosomal subunit blocking the translocational step in amino acid elongation, thus inhibiting protein synthesis (Schneider‐Poetsch et al. 2010). Emetine inhibits protein synthesis by binding onto the 40S subunit of ribosomes and inhibiting translocation of proteins (Akinboye and Bakare 2011). Puromycin acts as an analogue of the 3′‐terminal end of aminoacyl‐tRNA, which results in premature amino acid chain termination during translation of proteins (Azzam and Algranati 1973).

This study supports reported concerns over the inhibitors being too disruptive to normal cellular function to use to measure natural rates of protein turnover (Yewdell et al. 2011; Geva‐Zatorsky et al. 2012). In all cases, the CC_50_ concentration for the drugs in combination was lower than the corresponding IC_50_, suggesting that even in combination protein synthesis inhibition could not be studied in the absence of an effect on other cellular functions. These data suggest that inhibiting mechanisms of protein synthesis by pharmacological interference (even with lower concentration combinations) is not a physiologically appropriate method of measuring *k*_*deg*_ because all protein systems, including those involved in protein degradation pathways, are likely to be affected. In support of this, Dai et al. reported that cycloheximide could affect protein degradation by activating the AKT (protein kinase B) leading to downstream effects on the normal functioning of the ubiquitin proteasome degradation (UPD) pathway (Dai et al. 2013). In addition to the drugs disrupting protein degradation machinery, there have been reports of protein synthesis inhibitors actively inducing a range of protein mRNA production that also impact accuracies for calculating protein degradation rates downstream (Hattori and Gross 1995; Schuetz et al. 1995; Stordeur et al. 1995). It should be noted that the incubation time with the protein synthesis inhibitor drugs was for 72 h in the current study and as such, measurement of degradation for proteins with medium or long (over 72 h) half‐lives are likely to be particularly problematic. Further optimisations with shorter incubation periods may be possible for proteins with shorter *t*_1/2_ but robust optimisation will be required.

Protein synthesis inhibitors are commonly used for measuring protein degradation yet in previous studies, there has been little consideration for their cytotoxic effects and virtually none have optimised a specific concentration to use. Several studies have used cycloheximide at millimolar concentrations, which was much higher than the nontoxic concentration range found here (Pan and Haines 1999; Princiotta et al. 2003; Jeong et al. 2005; Xie et al. 2010; Majumder et al. 2012). MTT assays were used as the main method of measuring CC_50_ and the level of cytotoxicity across the four protein synthesis inhibitor drugs and their combinations. Since MTT assays specifically assess the formazan production pathway as a measure of cellular mitochondrial damage, other forms of cytotoxicity assays including GSH, ATP, and trypan blue exclusion, which assess other mechanisms of cytotoxicity, were carried out to validate the findings. Good agreement across assays and drug combinations was observed with the exception of puromycin, cycloheximide, and emetine in which higher cellular toxicity was detected in MTT than other assays. Despite GSH assays showing higher cell viability across the different drug combinations, it should be noted that GSH assays alone could not be used to predict the CC_50_ in this study because the results were in disagreement with the other cytotoxicity assays employed. A potential limitation is that protein binding was not assessed in this study. However, it should be recognised that protein binding would be expected to impact both cytotoxicity and protein synthesis inhibition by impacting free‐drug concentration. Thus, the ratio would not be expected to be different.

Earlier studies with actinomycin D and puromycin reported toxicity in HeLa cells at concentrations within the range investigated here. Studies by Sawicki and Godman (1971) showed that at 0.08 *μ*mol/L actinomycin D was sufficient to cause cell toxicity in HeLa cells, which is in agreement with the present findings. Dudani et al. (1988) proposed that puromycin caused cytotoxicity at 0.9 *μ*mol/L in human cell lines, including HeLa cells, which also agreed with the presented results. Dudani et al. also reported a 79.6% protein synthesis inhibition at 0.9 mmol/L in HeLa cells which further supports our findings that puromycin is cytotoxic at concentrations lower than those required for protein synthesis inhibition. Conversely, Yin Low et al. (2009) conducted cytotoxicity assays on emetine in Huh‐7 cells and reported over 90% cell viability at 10 *μ*mol/L which is much higher concentrations than those used here. Although the reason for this disparity is not apparent, cytotoxicity of these inhibitors may vary between different cell types. The single drug analyses were carried out in HepG2 and primary rat hepatocytes with reasonable agreement in protein synthesis inhibition and cytotoxicity for actinomycin D, emetine, and puromycin as shown in the linear relationship displayed in Figure 2. This study was carried out in readily available HepG2 cells and primary rat hepatocytes with the aim of transferring the optimised conditions onto primary human hepatocytes to validate a more physiologically accurate *k*_*deg*_ prediction (Wilkening et al. 2003). However, due to the presented findings, an alternative approach to *k*_*deg*_ determination is now being explored.

Despite the wide‐ranging importance of protein degradation, there has been no single recognised method for its measurement. However, these data indicate that the use of protein synthesis inhibitors should be avoided. The more recent methods of measuring rates of degradation focus on high‐throughput approaches aiming to quantify many different proteins in parallel; these involve metabolic labelling of proteins of interest followed by MS analysis (Doherty and Beynon 2006). Newly developed quantitative proteonomic methods provide an important alternative to chemical inhibition, however, reproducibility across different experiments and the impact of protein labelling on endogenous protein degradation warrants full investigation.

## Author Contributions

C.C wrote the manuscript. A.O, M.S, L.A, and C.C participated in experimental design. C.C conducted experiments with recommendations from P.M and N.J.L. Data was analysed by C.C, and A.O. L.A, P.M, N.J.L, M.S, and A.O contributed to the editing of the manuscript.

## Disclosure

The authors report no declaration of interest.
